# Supplementary material for: Eosinophils preserve bone homeostasis by inhibiting excessive osteoclast formation and activity via eosinophil peroxidase
Source: Nat Commun. 2024 Feb 5;15:1067. doi: 10.1038/s41467-024-45261-8 (PMC10844633; doi:10.1038/s41467-024-45261-8)
Supplement: Supplementary file 2 — Supplementary Information [file 41467_2024_45261_MOESM2_ESM.pdf]

Eosinophils preserve bone homeostasis by inhibiting excessive osteoclast formation and activity via eosinophil peroxidase

Supplementary Figure Legends

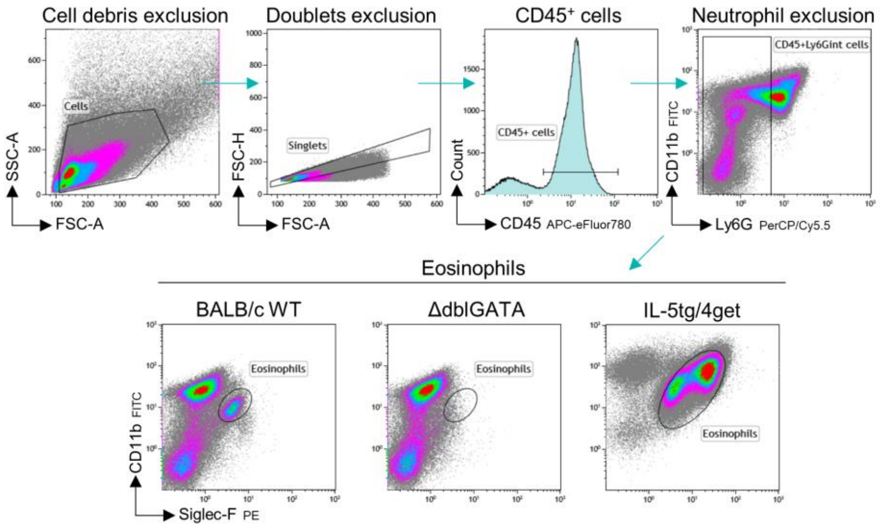

Sup. Fig. 1: Gating strategy of eosinophils in the bone marrow. Gating strategy of eosinophils (Siglec-F^{+}CD11b^{+}Ly6G^{int}CD45^{+}) in the bone marrow (BM) of wildtype (WT), eosinophil-deficient ΔdblGATA, and hyper-eosinophilic IL-5tg/ 4get mice.

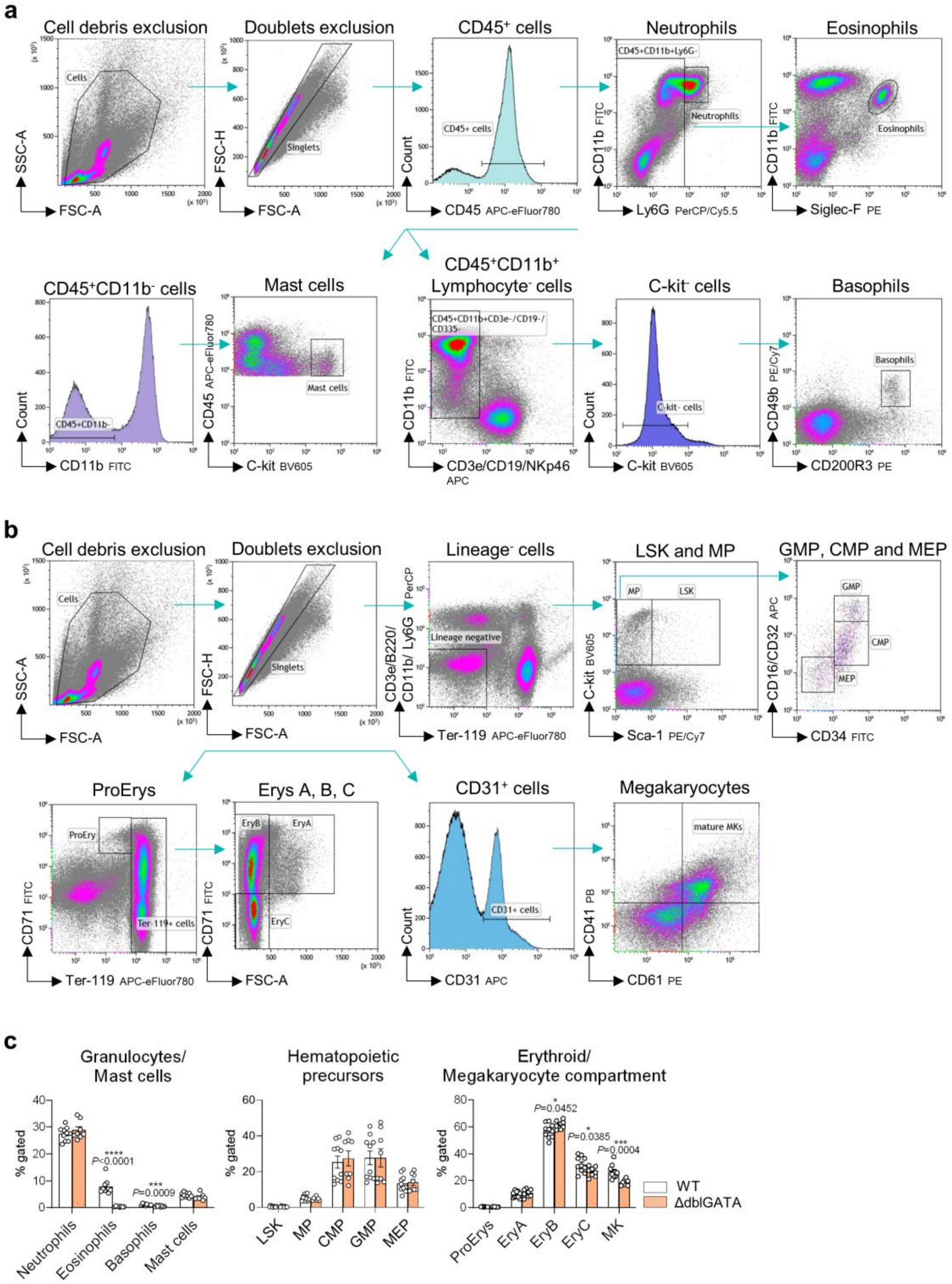

Sup. Fig. 2: Analysis of GATA-1-expressing lineages in WT vs. ΔdblGATA mice. a) Gating strategy of neutrophils (CD11b^{+}Ly6G^{high}CD45^{+}), eosinophils (Siglec-F^{+}CD11b^{+}Ly6G^{int}CD45^{+}), mast cells (C-kit^{+}CD11b^{-}CD45^{+}), and basophils (CD200R3^{+}CD49b^{+}C-kit^{-}lymphocyte^{-}CD11b^{+}CD45^{+}). **b)** Gating strategy of LSK (C-kit^{+}Sca-1^{+}lineage^{-}), multipontent progenitors (MP, C-kit^{+}Sca-1^{-}lineage^{-}), common myeloid progenitors (CMP, CD16/CD32^{int}CD34^{+}C-kit^{+}Sca-1^{-}lineage^{-}), granulocyte-macrophage progenitors (GMP, CD16/CD32^{high}CD34^{+}C-kit^{+}Sca-1^{-}lineage^{-}), megakaryocyte-erythroid progenitors (MEP, CD16/CD32^{-}CD34^{-}C-kit^{+}Sca-

1^-lineage$^-$), pro-erythroblasts (ProEry, $CD71^+$Ter-119^{int}), EryA erythroblasts ($CD71^+Ter119^+FSC^{high}$) EryB erythroblasts ($CD71^+Ter119^+FSC^{low}$), EryC erythroblasts ($CD71^-$ $Ter119^+FSC^{low}$), and megakaryocytes (MKs, $CD41^+CD61^+CD31^+$). **c)** Percentage distribution of the above mentioned cell populations in the bone marrow (BM) comparing WT with ΔdblGATA mice (n=10, 8). Data are shown as mean ± SEM. Symbols represent individual mice. *P* values were determined by two-tailed Mann-Whitney test (2c eosinophils) or unpaired two-tailed t test (2c basophils, EryB, EryC, MK) for single comparisons. Asterisks mark statistically significant difference (**P*<0.05, ****P*<0.001 and ****P<0.0001). Source data are provided as a Source Data file.

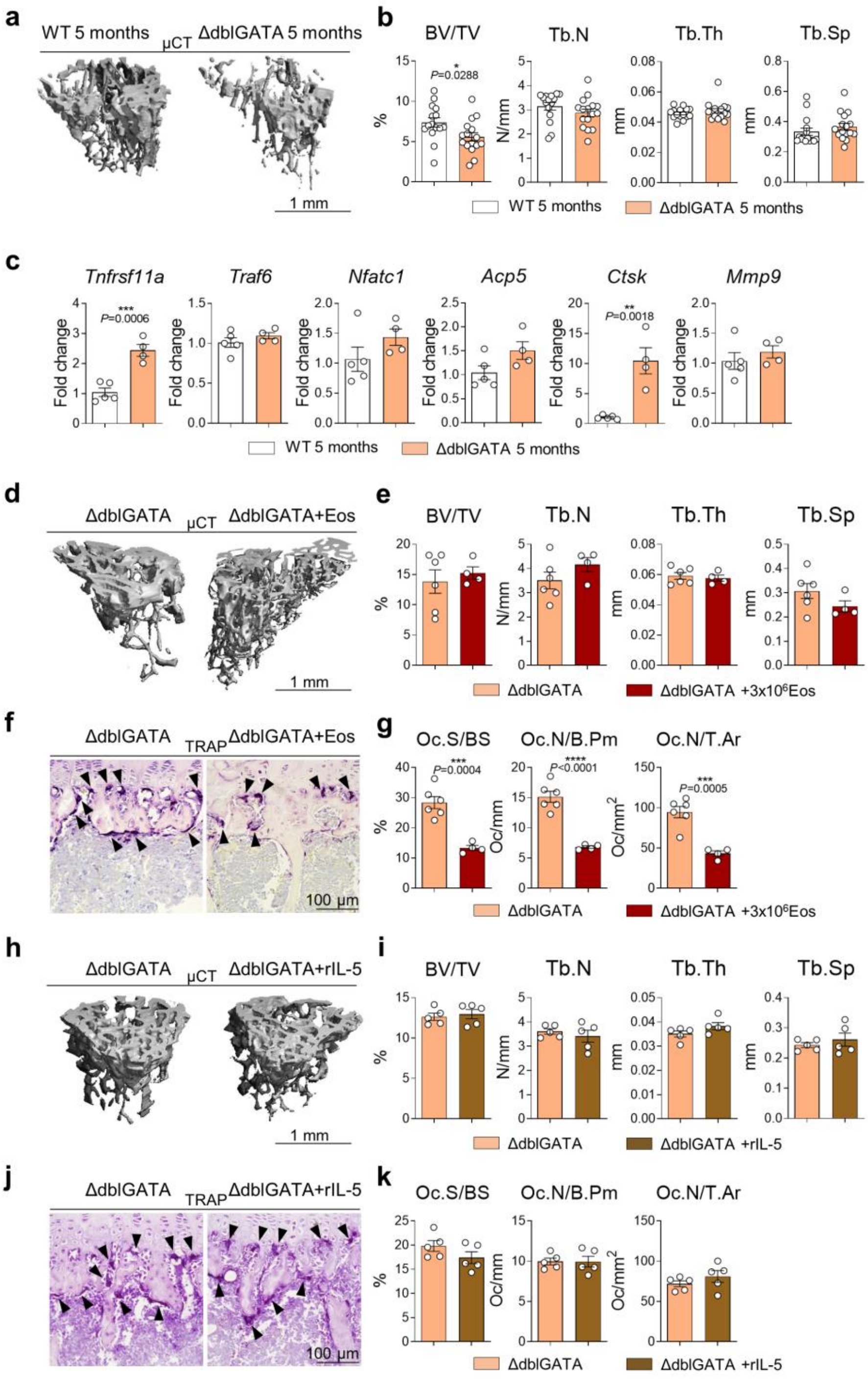

Sup. Fig. 3: Characterization of bone mass changes in ΔdblGATA mice at older age, as well as after reconstitution with eosinophils and IL-5 treatment. a, b) 3D trabecular structure by micro computed tomography (μCT) (a) and quantification of bone volume per total volume (BV/TV), trabecular number (Tb.N), trabecular thickness (Tb.Th), and trabecular separation (Tb.Sp) (b) in the tibial bone from 5 months old wildtype (WT) and ΔdblGATA mice (n=15, 16). Scale bar, 1 mm. **c)** mRNA expression of *Tnfrsf11a*, *Traf6*, *Nfatc1*, *Acp5*, *Ctsk*, and *Mmp9* in the long bone of 5 months old WT and ΔdblGATA mice (n=5, 4). **d, e)** 3D μCT trabecular structure (d) and quantification of BV/TV, Tb.N, Tb.Th, and Tb.Sp (e) in tibial bone

from ΔdblGATA mice and ΔdblGATA mice reconstituted with 3×10^6 eosinophils (n=6, 4). Scale bar, 1 mm. **f, g)** Representative tartrate-resistant acid phosphatase (TRAP) staining (f) and quantification of osteoclast surface per bone surface (Oc.S/BS), osteoclast number per bone perimeter (Oc.N/B.Pm), and osteoclast number per tissue area (Oc.N/T.Ar) (g) in tibial bone sections from ΔdblGATA mice and ΔdblGATA mice reconstituted with 3×10^6 eosinophils (n=6, 4). Triangles illustrate polynucleated osteoclasts. Scale bar, 100 µm. **h, i)** 3D µCT trabecular structure (h) and quantification of BV/TV, Tb.N, Tb.Th, and Tb.Sp (i) in tibial bone from ΔdblGATA mice and ΔdblGATA mice treated with 500 ng recombinant murine IL-5 every day for two consecutive weeks (n=5). Scale bar, 1 mm. **j, k)** Representative TRAP staining (j) and quantification of Oc.S/BS, Oc.N/B.Pm, and Oc.N/T.Ar (k) in tibial bone sections from ΔdblGATA mice and ΔdblGATA mice treated with 500 ng recombinant murine IL-5 every day for two consecutive weeks (n=5). Triangles illustrate polynucleated osteoclasts. Scale bar, 100 µm. Data are shown as mean ± SEM. Symbols represent individual mice. *P* values were determined by unpaired two-tailed t test (3b, 3c, 3g) for single comparisons. Asterisks mark statistically significant difference (**P*<0.05, ***P*<0.01, ****P*<0.001 and ****P<0.0001). Source data are provided as a Source Data file.

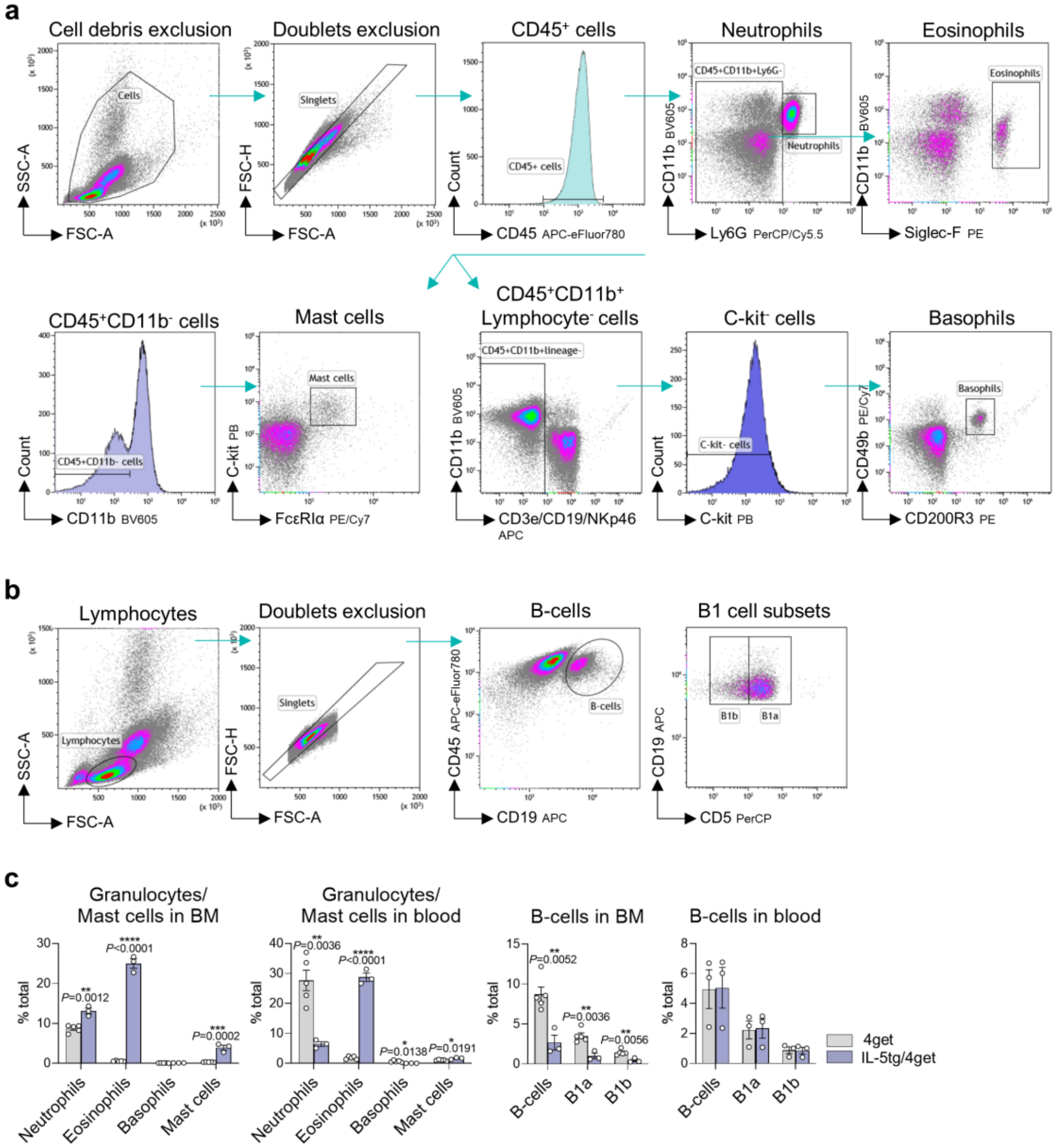

Sup. Fig. 4: Analysis of IL-5Rα-expressing immune cells in 4get vs. IL-5tg/ 4get mice. a) Gating strategy of neutrophils ($CD11b^+Ly6G^{high}CD45^+$), eosinophils ($Siglec\text{-}F^+CD11b^+Ly6G^{int}CD45^+$), mast cells ($Fc\varepsilon RI\alpha^+C\text{-}kit^+CD11b^-CD45^+$), and basophils ($CD200R3^+CD49b^+C\text{-}kit^-lymphocyte^-CD11b^+CD45^+$). **b)** Gating strategy of B-cells ($CD19^+CD45^+$), B1a ($CD5^+CD19^+CD45^+B220^-$) and B1b ($CD5^-CD19^+CD45^+B220^-$) cells. **c)** Percentage distribution of the above mentioned cell populations in the bone marrow (BM) (n=5, 3) and blood (n=5/ 3, 3) comparing 4get with IL-5tg/ 4get mice. Data are shown as mean ± SEM. Symbols represent individual mice. *P* values were determined by unpaired two-tailed t test (4c) for single comparisons. Asterisks mark statistically significant difference (**P*<0.05, ***P*<0.01, ****P*<0.001 and ****P<0.0001). Source data are provided as a Source Data file.

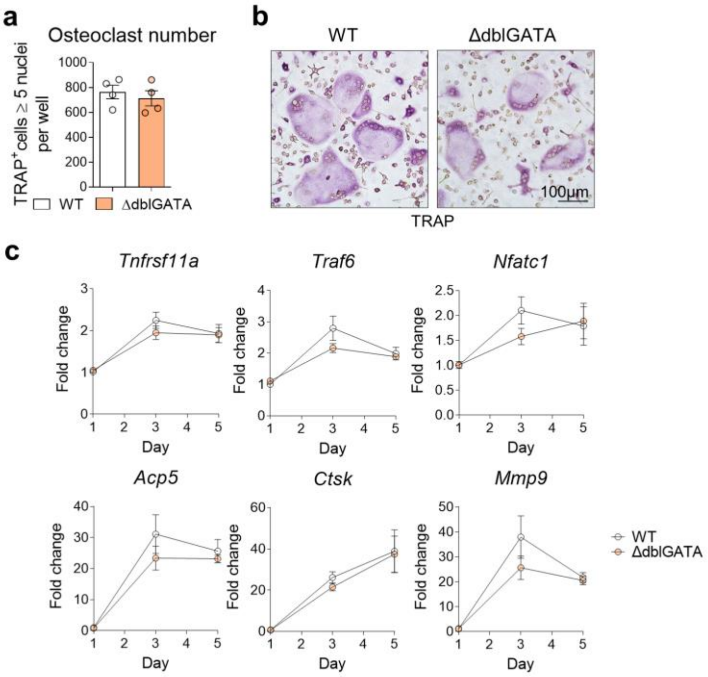

Sup. Fig. 5: Unchanged in vitro differentiation of osteoclasts from eosinophil-deficient ΔdblGATA mice. a, b) Quantification (a) and representative images (b) of TRAP-positive polynucleated (≥5 nuclei) osteoclasts from ΔdblGATA compared with wildtype (WT) mice at day 5 of differentiation (n=4). Scale bar, 100 µm. **c)** mRNA expression of *Tnfrsf11a*, *Traf6*, *Nfatc1*, *Acp5*, *Ctsk*, and *Mmp9* in cells from ΔdblGATA compared with WT mice during the course of osteoclastogenesis at day 1, 3, and 5 (n=6). Data are shown as mean ± SEM. Symbols represent individual mice. Source data are provided as a Source Data file.

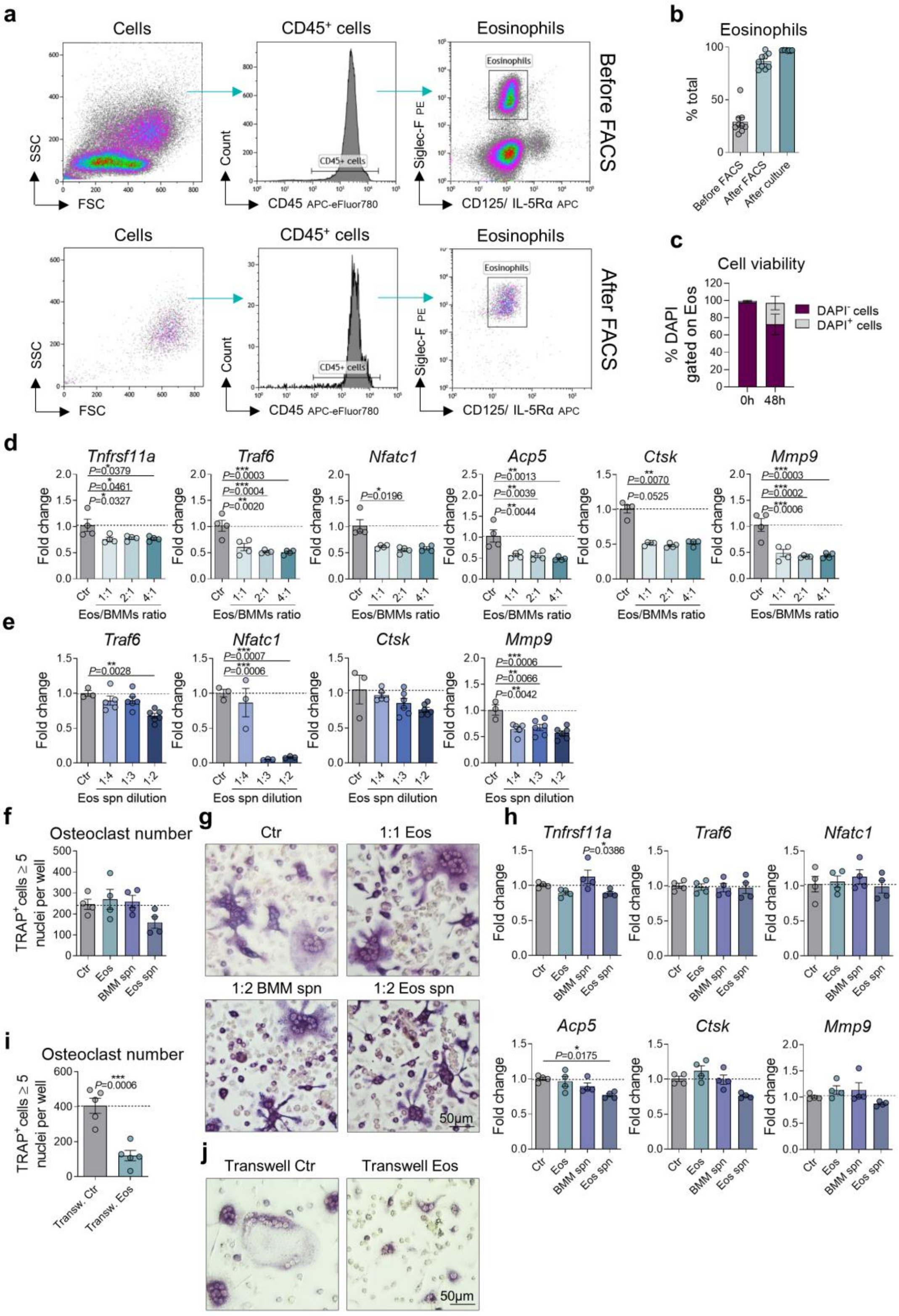

Sup. Fig. 6: Eosinophil isolation and different conditions of osteoclast stimultion with eosinophils and eosinophil supernatant. a, b) Sorting strategy for eosinophils (Siglec-F^{+}CD125^{int}CD45^{+} cells) from IL-5tg/ 4get mice (a) and purity (b) before fluorescence activated cell sorting (FACS), after FACS, and after additional culture for 48 hours (n=9, 9, 8). **c)** Flow cytometric analysis of eosinophil viability with DAPI comparing cells freshly after sorting with

eosinophils cultured for 48 hours (n=3, 3, 5, 5). **d)** mRNA expression of *Tnfrsf11a*, *Traf6*, *Nfatc1*, *Acp5*, *Ctsk*, and *Mmp9* in WT osteoclasts, co-cultured with different ratios of eosinophils (Eos) on day 0 for 48 hours compared with unstimulated control (n=4). **e)** mRNA expression of *Traf6*, *Nfatc1*, *Ctsk*, and *Mmp9* in WT osteoclasts, co-cultured with different amounts of eosinophil supernatant (Eos spn) on day 0 for 48 hours compared with unstimulated control (n=3, 5, 6, 6). **f, g)** Quantification (f) and representative images (g) of TRAP-positive polynucleated (≥5 nuclei) WT osteoclasts following culture with eosinophils (1:1 Eos/BMMs ratio), BMM supernatant (1:2 dilution), and eosinophil supernatant (1:2 dilution) on day 2 for 48 hours compared with unstimulated control (n=4). Scale bar, 50 µm. **h)** mRNA expression of *Tnfrsf11a*, *Traf6*, *Nfatc1*, *Acp5*, *Ctsk*, and *Mmp9* in WT osteoclasts following culture with eosinophils (1:1 Eos/BMMs ratio), BMM supernatant (1:2 dilution), and eosinophil supernatant (1:2 dilution) on day 2 for 48 hours compared with unstimulated control (n=4). **i, j)** Quantification (i) and representative images (j) of TRAP-positive polynucleated (≥5 nuclei) WT osteoclasts following culture with eosinophils (1:1 Eos/BMMs ratio) separated by a membrane with a pore size of 1µm on day 0 for 48 hours compared with transwell control (n=5). Scale bar, 50 µm. Data are shown as mean ± SEM. Symbols represent individual mice. *P* values were determined by unpaired two-tailed t test (6i) for single comparisons and Kruskal-Wallis Dunn′s test (6d Nfatc1, Ctsk, 6h Acp5), one-way ANOVA Dunnett′s test (6d Tnfrsf11a, Traf6, Acp5, Mmp9, 6e Traf6, Nfatc1, Mmp9) or one-way ANOVA Tukey′s test (6h Tnfrsf11a) for multiple comparisons. Asterisks mark statistically significant difference (**P*<0.05, ***P*<0.01 and ****P*<0.001). Source data are provided as a Source Data file.

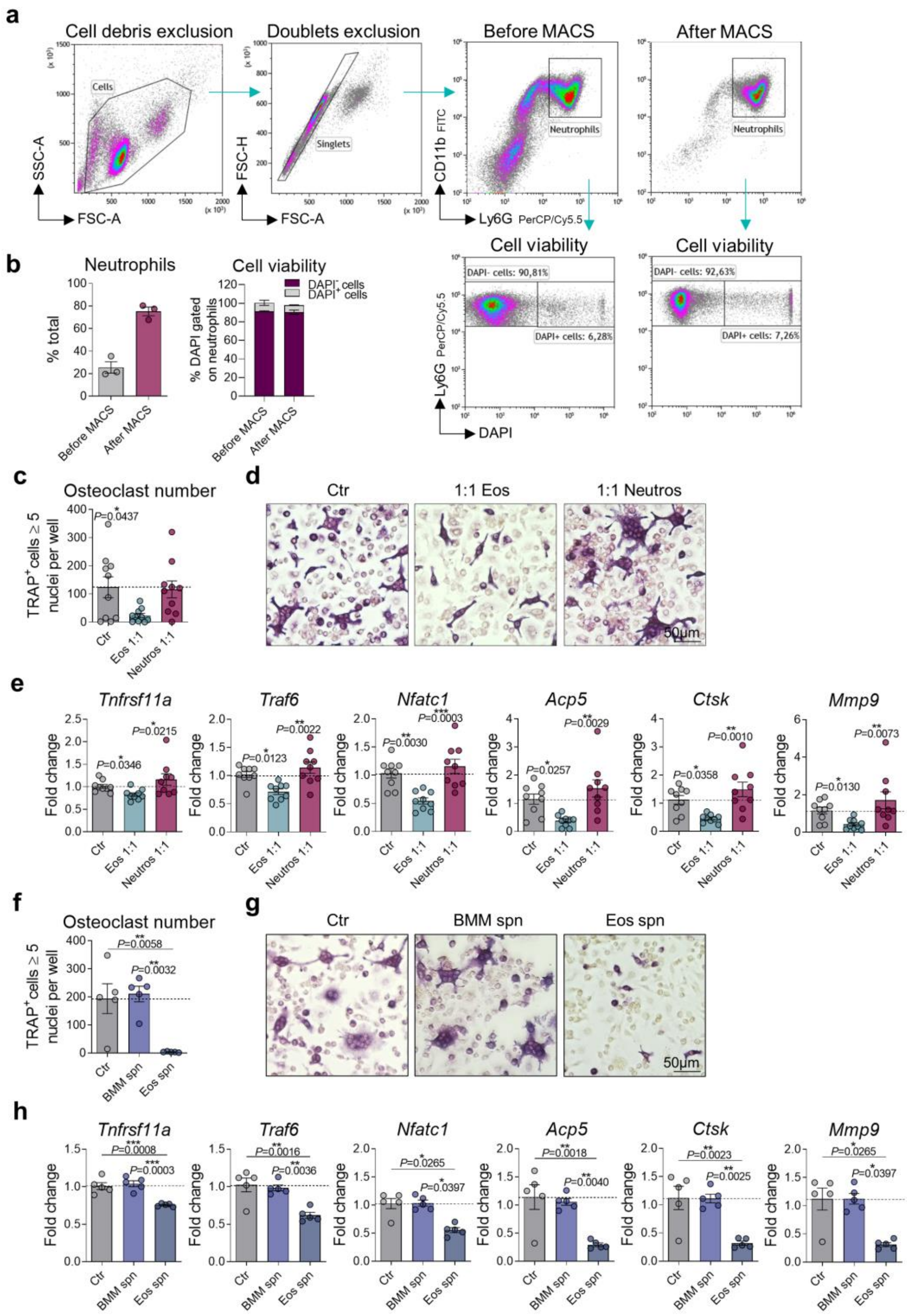

Sup. Fig. 7: Control conditions for the stimulation of osteoclasts with eosinophils and eosinophil supernatant. a, b) Gating strategy (a), purity, and viability analysis with DAPI (b) of neutrophils (Ly6G^{high}CD11b^{+}CD45^{+} cells) before and after negative selection by magnetic cell separation (MACS) (n=3). **c, d)** Quantification (c) and representative images (d) of TRAP-positive polynucleated (≥5 nuclei) WT osteoclasts following culture with eosinophils (1:1 Eos/BMMs ratio) or neutrophils (1:1 Neutros/BMMs ratio) on day 0 for 48 hours compared with unstimulated control (n=10). Scale bar, 50 μm. **e)** mRNA expression of *Tnfrsf11a*, *Traf6*,

Nfatc1, *Acp5*, *Ctsk*, and *Mmp9* in WT osteoclasts following culture with eosinophils (1:1 Eos/BMMs ratio) or neutrophils (1:1 Neutros/BMMs ratio) on day 0 for 48 hours compared with unstimulated control (n=9). **f, g)** Quantification (f) and representative images (g) of TRAP-positive polynucleated (≥5 nuclei) WT osteoclasts following culture with eosinophil supernatant (1:2 dilution) on day 0 for 48 hours compared with supernatant from BMMs (BMM spn, 1:2 dilution) and unstimulated control (n=5). Scale bar, 50 µm. **h)** mRNA expression of *Tnfrsf11a*, *Traf6*, *Nfatc1*, *Acp5*, *Ctsk*, and *Mmp9* in WT osteoclasts following culture with eosinophil supernatant (1:2 dilution) on day 0 for 48 hours compared with supernatant from BMMs (BMM spn, 1:2 dilution) and unstimulated control (n=5). Data are shown as mean ± SEM. Symbols represent individual mice. *P* values were determined by Kruskal-Wallis Dunn′s test (7e Tnfrsf11a, Traf6, Acp5, Mmp9, 7h Nfatc1, Mmp9) or one-way ANOVA Tukey′s test (7c, 7e Nfatc1, Ctsk, 7f, 7h Tnfrsf11a, Traf6, Acp5, Ctsk) for multiple comparisons. Asterisks mark statistically significant difference (*P<0.05, **P<0.01 and ***P<0.001). Source data are provided as a Source Data file.

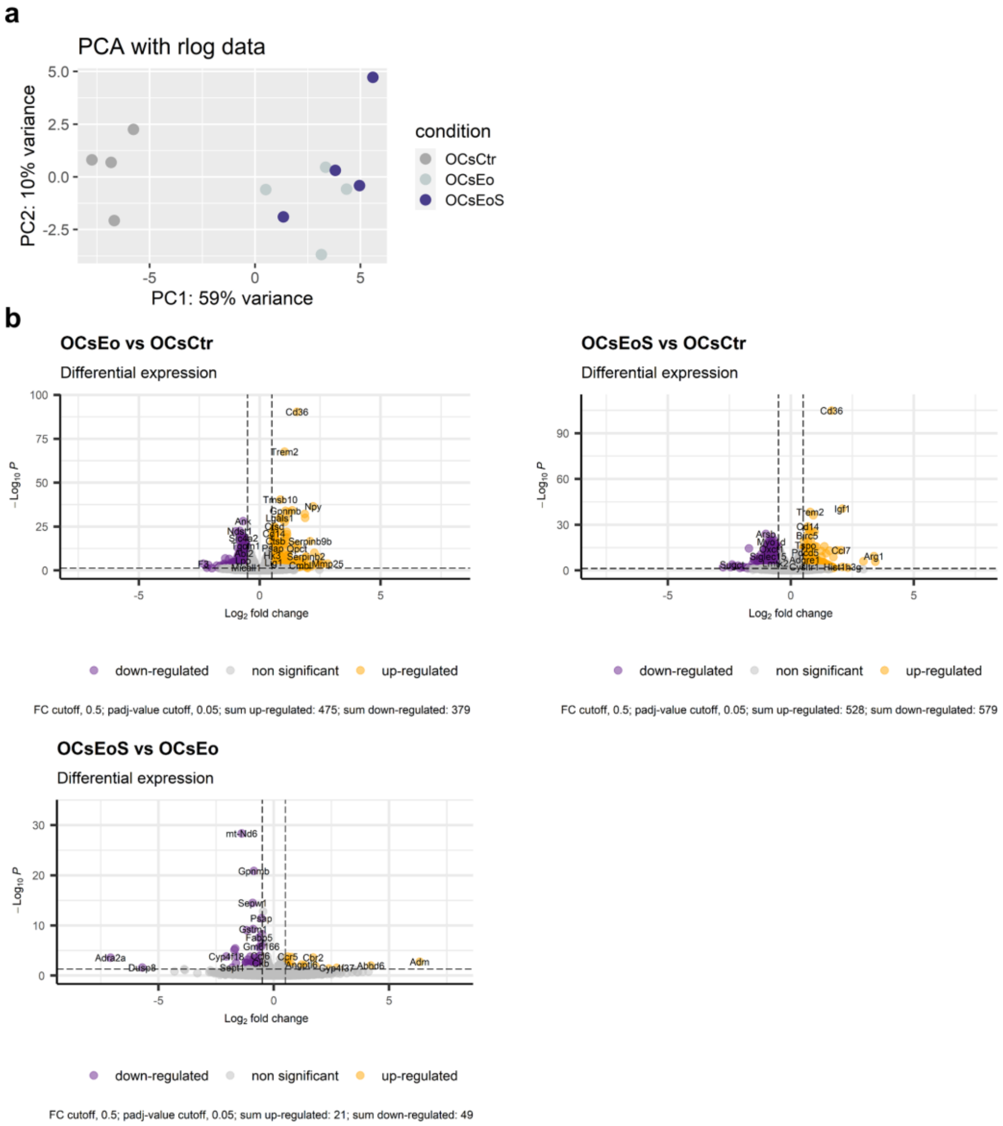

Sup. Figure 8: Bulk RNA-sequencing analysis after exclusion of eosinophil-associated genes. Bulk RNA-seq. analysis on WT osteoclasts stimulated with eosinophils (2:1 Eos/BMMs ratio; OCsEo) or eosinophil supernatant (1:2 dilution; OCsEoS) for 48 hours as compared with the unstimulated control (OCsCtr) group (n=4). **a)** Principal component analysis (PCA) was used to visualize the distinct patterns among individual samples of the three groups, when eosinophil-associated genes were excluded. **b)** Volcano plots were generated to show the differentially expressed genes (DEGs) between the groups OCsEo vs. OCsCtr, OCsEoS vs. OCsCtr, and OCsEoS vs. OCsEo, with the exclusion of eosinophil-associated genes. Genes with a log2 fold change greater than 0.5 and an adjusted P value (Padj) less than 0.05 were considered differentially expressed.

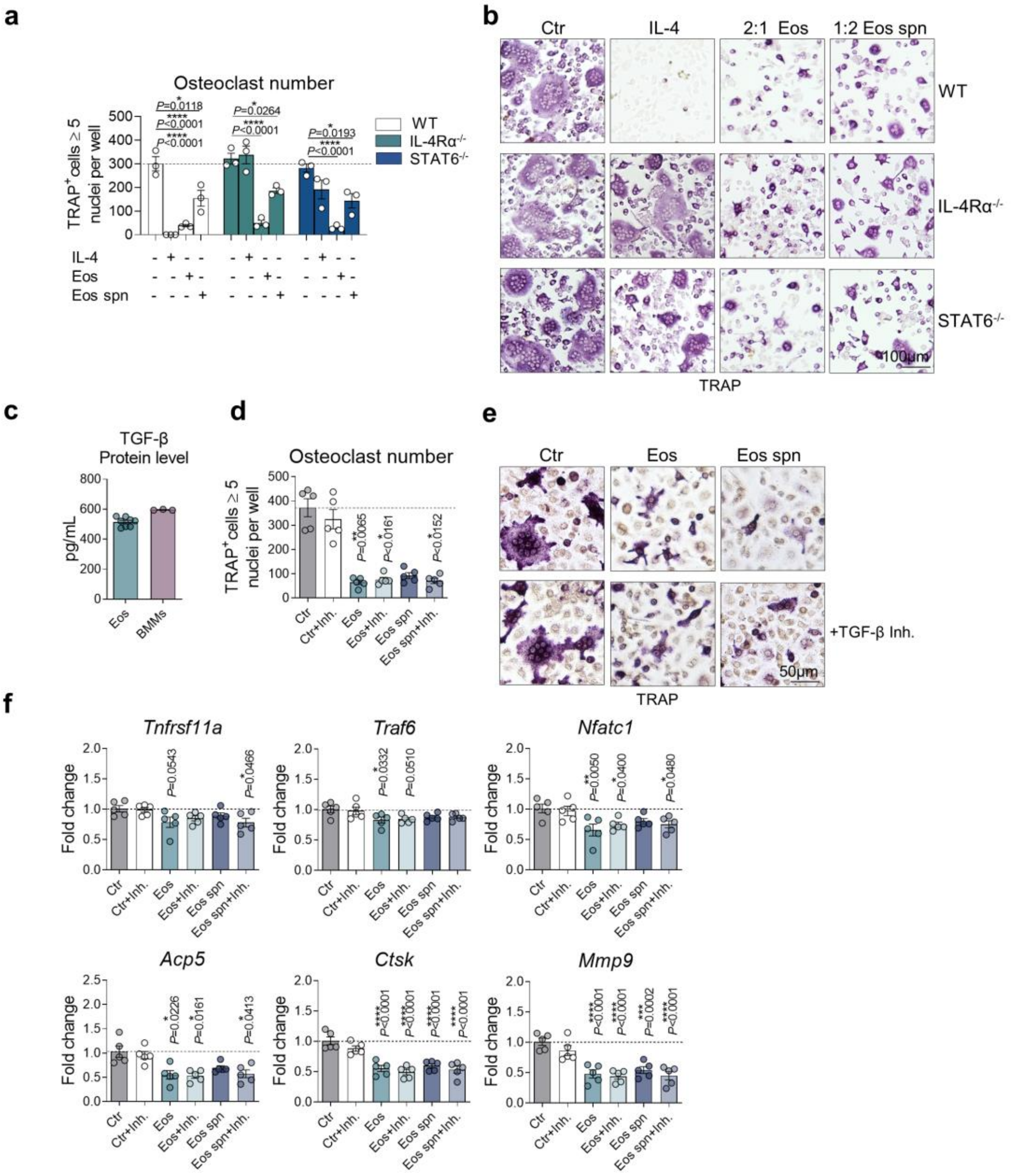

Sup. Figure 9: Osteoclast inhibition by eosinophils is IL-4/ IL-13 and TGF-β independent. a, b) Quantification (a) and representative images (b) of TRAP-positive polynucleated (≥5 nuclei) wildtype (WT), IL-4Rα$^{-/-}$, and STAT6$^{-/-}$ osteoclasts co-cultured with 10 ng/mL recombinant IL-4, eosinophils (2:1 Eos/BMMs ratio), and eosinophil supernatant (1:2 dilution) on day 0 of culture for 48 hours compared with unstimulated control (n=3). Scale bar, 100 µm. **c)** Protein level of TGF-β in the supernatant of eosinophils and BMMs after culture for 48 hours (n=8, 3). **d, e)** Quantification (d) and representative images (e) of TRAP-positive polynucleated (≥5 nuclei) WT osteoclasts following culture with eosinophils (1:1 Eos/BMMs ratio) and eosinophil supernatant (1:2 dilution) on day 0 for 48 hours compared with unstimulated control without or with 0.1 µM TGF-β receptor inhibitor Galunisertib (n=5). Scale bar, 50 µm. **f)** mRNA expression of *Tnfrsf11a*, *Traf6*, *Nfatc1*, *Acp5*, *Ctsk*, and *Mmp9* in WT osteoclasts following culture with eosinophils (1:1 Eos/BMMs ratio) and eosinophil supernatant (1:2 dilution) on day

0 for 48 hours compared with unstimulated control without or with 0.1 µM TGF-β receptor inhibitor Galunisertib (n=5). Data are shown as mean ± SEM. Symbols represent individual mice. *P* values were determined by Kruskal-Wallis Dunn′s test (9d, 9f Acp5), one-way ANOVA Dunnett′s test (9f Tnfrsf11a, Traf6, Nfatc1, Ctsk, Mmp9) or two-way ANOVA Tukey′s test (9a) for multiple comparisons. Asterisks mark statistically significant difference (*$P<0.05$, **$P<0.01$, ***$P<0.001$ and **** $P<0.0001$). Source data are provided as a Source Data file.

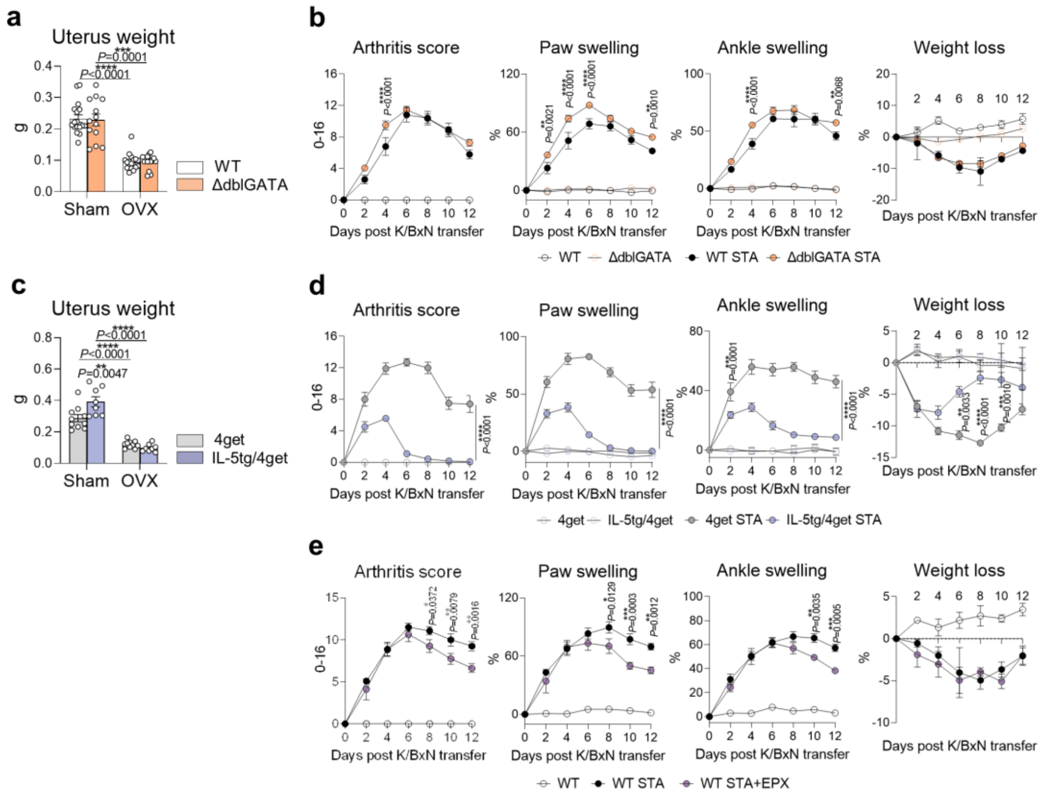

Sup. Fig. 10: Measurement of ovariectomy and arthritis-mediated disease parameters in ΔdblGATA mice, IL-5tg/ 4get mice, and BALB/c WT mice treated with EPX. a) Uteri weight of wildtype (WT) and ΔdblGATA mice 6 weeks after sham surgery vs. ovariectomy (OVX) (n=17, 13, 17, 15). **b)** Arthritis score, proportional paw swelling, ankle swelling, and weight loss of WT and ΔdblGATA mice during the course of serum transfer arthritis (STA) (n=5, 9, 5, 9). **c)** Uteri weight of 4get and IL-5tg/ 4get mice 6 weeks after sham surgery vs. OVX (n=10, 8, 10, 8). **d)** Arthritis score, proportional paw swelling, ankle swelling, and weight loss of 4get and IL-5tg/ 4get mice during the course of STA (n=5, 6, 5, 6). **e)** Arthritis score, proportional paw swelling, ankle swelling, and weight loss of WT, STA, and STA+EPX mice during the course of STA (n=5, 6, 4). Data are shown as mean ± SEM. Symbols represent individual mice. *P* values were determined by Kruskal-Wallis Dunn′s test (10a) or two-way ANOVA Tukey′s test (10b, 10c, 10d, 10e) for multiple comparisons. Asterisks mark statistically significant difference (**P*<0.05, ***P*<0.01, ****P*<0.001 and **** *P*<0.0001). Source data are provided as a Source Data file.

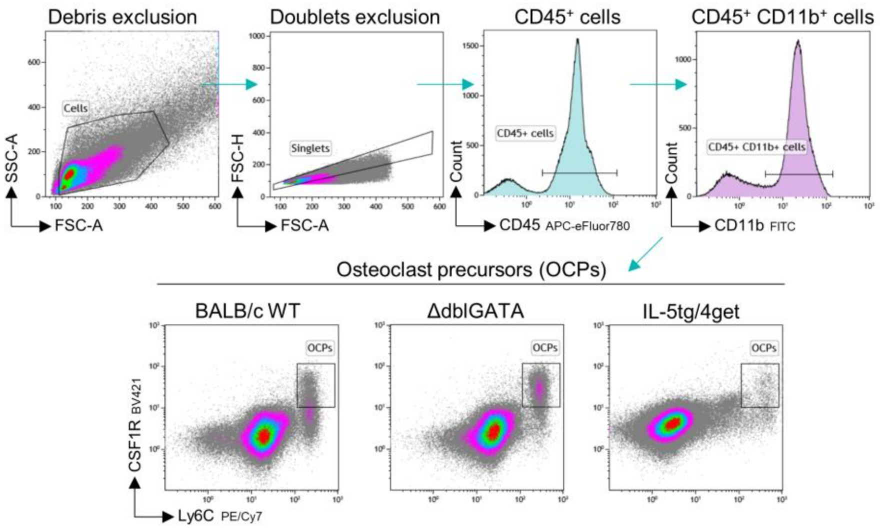

Sup. Fig. 11: Gating strategy of osteoclast precursors in the bone marrow. Gating strategy of osteoclast precursors (OCPs/ CSF1R^{+}Ly6C^{high}CD11b^{+}CD45^{+} cells) in the bone marrow (BM) of wildtype (WT), eosinophil-deficient ΔdblGATA, and hyper-eosinophilic IL-5tg/ 4get mice.
