# Supplementary material for: Eosinophils preserve bone homeostasis by inhibiting excessive osteoclast formation and activity via eosinophil peroxidase
Source: Nat Commun. 2024 Feb 5;15:1067. doi: 10.1038/s41467-024-45261-8 (PMC10844633; doi:10.1038/s41467-024-45261-8)
Supplement: Supplementary file 3 — Reporting Summary [file 41467_2024_45261_MOESM3_ESM.pdf]

nature portfolio

Corresponding author(s): Aline Bozec
Darja Andreev

Last updated by author(s): Jan 17, 2024

Reporting Summary

Nature Portfolio wishes to improve the reproducibility of the work that we publish. This form provides structure for consistency and transparency in reporting. For further information on Nature Portfolio policies, see our Editorial Policies and the Editorial Policy Checklist.

Statistics

For all statistical analyses, confirm that the following items are present in the figure legend, table legend, main text, or Methods section.

n/a	Confirmed	
☐	☒	The exact sample size (*n*) for each experimental group/condition, given as a discrete number and unit of measurement
☐	☒	A statement on whether measurements were taken from distinct samples or whether the same sample was measured repeatedly
☐	☒	The statistical test(s) used AND whether they are one- or two-sided<br>*Only common tests should be described solely by name; describe more complex techniques in the Methods section.*
☐	☒	A description of all covariates tested
☐	☒	A description of any assumptions or corrections, such as tests of normality and adjustment for multiple comparisons
☐	☒	A full description of the statistical parameters including central tendency (e.g. means) or other basic estimates (e.g. regression coefficient) AND variation (e.g. standard deviation) or associated estimates of uncertainty (e.g. confidence intervals)
☐	☒	For null hypothesis testing, the test statistic (e.g. *F*, *t*, *r*) with confidence intervals, effect sizes, degrees of freedom and *P* value noted<br>*Give P values as exact values whenever suitable.*
☒	☐	For Bayesian analysis, information on the choice of priors and Markov chain Monte Carlo settings
☒	☐	For hierarchical and complex designs, identification of the appropriate level for tests and full reporting of outcomes
☒	☐	Estimates of effect sizes (e.g. Cohen's *d*, Pearson's *r*), indicating how they were calculated

Our web collection on statistics for biologists contains articles on many of the points above.

Software and code

Policy information about availability of computer code

Data collection: Human and mouse data were collected as described below.

Data analysis: Bulk RNA-seq. was carried out by Novogene (UK). As described by novogene: A total amount of 1 µg RNA per sample was used as input material for the RNA sample preparations. Sequencing libraries were generated using NEBNext® Ul-traTM RNA Library Prep Kit for Illumina® (NEB, Cat# E7770) following manufacturer's recommendations and index codes were added to attribute sequences to each sample. The clustering of the index-coded samples was performed according to the manufacturer's instructions. After cluster generation, the library preparations were sequenced on an Illumina platform and paired-end reads were generated. Clean reads were obtained by removing reads containing adapter, reads containing poly-N, and low-quality reads from raw data. All the downstream analyses were based on the clean data with high quality. The filtered reads were aligned to the reference genome (mm10) with Hisat2 v2.0.5. Feature Counts v1.5.0-p3 was used to count the reads numbers mapped to each gene.
Following key packages were used for further analysis with these read counts:
BiocManager https://bioconductor.github.io/BiocManager/ 1.30.22
DESeq2 https://bioconductor.org/packages/DESeq2/ 1.34.0
EnhancedVolcano https://bioconductor.org/packages/EnhancedVolcano/ 1.12.0
glmpca https://cran.r-project.org/web/packages/glmpca/index.html 0.2.0
genefilter https://bioconductor.org/packages/genefilter 1.76.0
pheatmap https://cran.r-project.org/web/packages/pheatmap/index.html 1.0.12

All statistical analyses of human and mouse experiments were performed using Graph-Pad Prism-Software 9.

For manuscripts utilizing custom algorithms or software that are central to the research but not yet described in published literature, software must be made available to editors and reviewers. We strongly encourage code deposition in a community repository (e.g. GitHub). See the Nature Portfolio guidelines for submitting code & software for further information.

Data

Policy information about availability of data

All manuscripts must include a data availability statement. This statement should provide the following information, where applicable:

- Accession codes, unique identifiers, or web links for publicly available datasets
- A description of any restrictions on data availability
- For clinical datasets or third party data, please ensure that the statement adheres to our policy

The bulk RNA-sequencing data generated in this study were aligned to the reference genome GRCm38/mm10 [https://www.ncbi.nlm.nih.gov/datasets/genome/GCF_000001635.20/] and have been deposited in the Gene Expression Omnibus (GEO) database under accession code “GSE203541 [https://www.ncbi.nlm.nih.gov/geo/query/acc.cgi?acc=GSE203541]”. Source data are provided with this paper containing all raw data.

Research involving human participants, their data, or biological material

Policy information about studies with human participants or human data. See also policy information about sex, gender (identity/presentation), and sexual orientation and race, ethnicity and racism.

Reporting on sex and gender	Rheumatoid arthritis (RA) primarily impacts females, with a female-to-male ratio of 3:1. As a result, the patient group comprises a higher proportion of women compared to men.
Reporting on race, ethnicity, or other socially relevant groupings	No social categorization was established.
Population characteristics	Demographic characteristics (age, sex and body mass index), disease-specific characteristics (disease duration and DAS 28 score), bone parameters (trabecular and cortical bone vol-ume), and eosinophil parameters (eosinophil counts and ECP levels) of the healthy controls and RA patients are listed in table 1.
Recruitment	Patients and healthy donors were enrolled by the Department of Internal Medicine 3 – Rheumatology and Immunology, Friedrich-Alexander-University (FAU) Erlangen-Nürnberg and Universitätsklinikum Erlangen, Erlangen, Germany. In order to ensure a uniform participant cohort and minimize the impact of treatment variables, we selected RA patients that received methotrexate monotherapy (early RA), with no treatment with other conventional synthetic, targeted synthetic, or biologic disease-modifying anti-rheumatic drugs (DMARDs). Furthermore, patients receiving additional glucocorticoid treatment were separately analyzed from RA patients receiving no glucocorticoid treatment. Healthy controls were sex and age matched. For the blood donation, participants received financial compensation.
Ethics oversight	Healthy controls and RA patients provided written informed consent to participate in the present study. All analyses were approved by the institutional review board (IRB) of the University Clinic Erlangen and were in accordance with the Declaration of Helsinki–Ethical Principles for Med-ical Research Involving Human Subjects.

Note that full information on the approval of the study protocol must also be provided in the manuscript.

Field-specific reporting

Please select the one below that is the best fit for your research. If you are not sure, read the appropriate sections before making your selection.

☒ Life sciences ☐ Behavioural & social sciences ☐ Ecological, evolutionary & environmental sciences

For a reference copy of the document with all sections, see nature.com/documents/nr-reporting-summary-flat.pdf

Life sciences study design

All studies must disclose on these points even when the disclosure is negative.

Sample size	The sample size used in the experiments followed the common practice of the field (Adam, S. et al. JAK inhibition increases bone mass in steady-state conditions and ameliorates pathological bone loss by stimulating osteoblast function. Sci Transl Med 12 (2020)) and was verified by G*Power (version 3.1.9.7).
Data exclusions	In all experiments all measured values that met Quality Control criteria (e.g. correct genotype, induced disease model, effective staining, gene expression) were included and statistically analysed. This exclusion criteria was predetermined.
Replication	All experimental findings were reliably reproduced at least 3 times and mostly pooled. In some instances, representative experiments were displayed.
Randomization	Mice were randomly assigned to experimental groups, which were distributed among cages to compensate for covariates.
Blinding	Investigation of all murine and human samples was performed in a blinded manner.

Reporting for specific materials, systems and methods

We require information from authors about some types of materials, experimental systems and methods used in many studies. Here, indicate whether each material, system or method listed is relevant to your study. If you are not sure if a list item applies to your research, read the appropriate section before selecting a response.

Materials & experimental systems

n/a	Involved in the study
☐	☒ Antibodies
☒	☐ Eukaryotic cell lines
☒	☐ Palaeontology and archaeology
☐	☒ Animals and other organisms
☒	☐ Clinical data
☒	☐ Dual use research of concern
☒	☐ Plants

Methods

n/a	Involved in the study
☒	☐ ChIP-seq
☐	☒ Flow cytometry
☒	☐ MRI-based neuroimaging

Antibodies

Antibodies used

1. Primary antibodies for IF staining for the following antigen (clone), from the stated source with appropriate Cat#:

Anti-Epx antibody (clone AHE-1); Abcam; Cat#ab190715
Anti-Epx antibody (polyclonal); Abcam; Cat#ab65319
Anti-CD68 antibody (clone FA-11); GeneTex; Cat#GTX43914
Alexa Fluor® 647 Rat Anti-Mouse Siglec-F (clone E50-2440); BD Pharmingen; Cat#562680
Alexa Fluor® 647 anti-mouse/rat CD61 Antibody (clone 2C9.G2); BioLegend; Cat#104314

2. Secondary antibodies for IF staining from the following host, for the following antigen (attached fluorophore), from the stated source with appropriate Cat#:

Goat anti-mouse IgG H&L (Alexa Fluor®555); Abcam; Cat#ab150114
Alexa Fluor®488 Streptavidin; Biolegend; Cat# 405235
Goat anti-Rabbit IgG (H+L) Cross-Adsorbed Secondary Antibody (Alexa Fluor™ 488); Invitrogen; Cat#A-11008

3. Flow cytometry antibodies against the following antigen (clone) with the following fluorophore and from the stated source with appropriate Cat#:

anti-CD45 APC-eFluor™ 780 (clone 30-F11); eBioscience; Cat#47-0451-82
anti-CD45 PerCP/ Cyanine 5.5 (clone 30-F11); BioLegend; Cat#103132
anti-CD11b FITC (clone M1/70); BD Pharmingen; Cat#557396
anti-CD11b PerCP (clone M1/70); BioLegend; Cat#101230
anti-CD11b Brilliant Violet 605™ (M1/70); BioLegend; Cat#101257
anti-Ly6G PerCP/ Cyanine 5.5 (clone 1A8); BioLegend; Cat#127616
anti-Ly6C PE/ Cyanine 7 (clone HK1.4); BioLegend; Cat#128018
anti-CD115 (CSF-1R) Brilliant Violet 421™ (clone AFS98); BioLegend; Cat#135513
anti-Siglec-F PE (clone E50-2440); BD Pharmingen; Cat#562068
anti-CD117 (c-Kit) Pacific Blue™ (clone 2B8); BioLegend; Cat#105820
anti-CD117 (c-Kit) Brilliant Violet 605™ (clone 2B8); BioLegend; Cat#105847
anti-CD49b PE/ Cyanine 7 (clone DX5); BioLegend; Cat#108922
anti-FcεRIα PE/ Cyanine 7 (clone MAR-1); BioLegend; Cat#134318
anti-CD200R3 PE (clone Ba13); BioLegend; Cat#142206
anti-CD3ε PE/ Cyanine5 (clone 145-2C11); BioLegend; Cat#100310
anti-CD3ε APC (clone 145-2C11); BioLegend; Cat#100312
anti-CD19 APC (clone 1D3); BD Pharmingen; Cat#550992
anti-CD335 (NKp46) APC (clone 29A1.4); BioLegend; Cat#137608
anti-CD31 Alexa Fluor® 647 (clone MEC13.3); BioLegend; Cat#102516
anti-CD61 PE (clone 2C9.G2); BioLegend; Cat#104308
anti-CD41 Pacific Blue™ (clone MWReg30); BioLegend; Cat#133932
anti-Ly-6A/E (Sca-1) PE/ Cyanine 7 (clone E13-161.7); BioLegend; Cat#122514
anti-CD16/CD32 APC (clone 93); eBioscience; Cat#17-0161-82
anti-CD34 FITC (clone RAM34); BD Pharmingen; Cat#560238
anti-CD45R/B220 PerCP (clone RA3-6B2); BD Pharmingen; Cat#553093
anti-CD45R/B220 Brilliant Violet 510™ (clone RA3-6B2); BioLegend; Cat#103247
anti-TER-119 APC-efluor™ 780 (clone TER-119); eBioscience; Cat#47-5921-82
anti-CD71 FITC (clone RI7217); BioLegend; Cat#113806
anti-CD5 PerCP (clone 53-7.3); BD Pharmingen; Cat#553025
anti-CD125 (IL-5Rα) APC (clone REA343); Miltenyi Biotec; Cat#130-118-561

Validation

The antibodies selected for use are exclusively well-established and validated clones, with validation conducted by the

Validation

manufacturers. To guarantee the delivery of high-quality products, the antibodies are validated by the manufacturers through various processes. This includes testing on knockout (KO) or knockdown (KD) cell lysates, utilizing CRISPR/Cas9 or siRNA, along with negative control cell lines to confirm antibody specificity. The evaluation extends to multiple species (e.g. human, mouse, rat) when applicable, ensuring a comprehensive assessment of antibody cross-reactivity. Furthermore, side-by-side comparative testing is conducted with competitor antibodies to verify sensitivity. Internal controls are employed for the same purpose across different lots, ensuring a consistent performance from lot to lot. Clones initially designed for a specific application, such as WB or ChIP, undergo additional validation in multiple applications like microscopy and flow cytometry. This approach provides users with the flexibility to supplement their research with additional assays. Additionally, manufacturers optimize the suggested use of the antibody by providing a titration range, enhancing the adaptability and effectiveness of the product in various experimental settings.

For detailed information, please refer to the respective product page for each antibody, corresponding to the provided catalog number.

1. Primary antibodies for IF staining (antigen, clone, source, Cat#) were used in the following dilutions:

Anti-Epx antibody (clone AHE-1); Abcam; Cat#ab190715; 1:200; validated by the manufacturer for IF staining of human tissue; titrated inhouse to define the optimal dilution (1:50; 1:100; 1:200; 1:500; 1:800)

Anti-Epx antibody (polyclonal); Abcam; Cat#ab65319; 1:100; validated inhouse for reactivity against mouse and optimal dilution (1:50; 1:100; 1:200; 1:500; 1:800), the assessment involved comparing IF staining in wild-type bone marrow with that of eosinophil knockout mice

Anti-CD68 antibody (clone FA-11); GeneTex; Cat#GTX43914; 1:100; validated by the manufacturer for IF staining of human tissue; titrated inhouse to define the optimal dilution (1:50; 1:100; 1:200; 1:500; 1:800)

Alexa Fluor® 647 Rat Anti-Mouse Siglec-F (clone E50-2440); BD Pharmingen; Cat#562680; 5 µg/ mouse; antibody amount was tested in a rage from 1 µg - 10 µg/ mouse according to utilization in previous publication DOI: 10.1681/ASN.2016020232

Alexa Fluor® 647 anti-mouse/rat CD61 Antibody (clone 2C9.G2); BioLegend; Cat#104314; 1:100; validated by the manufacturer for IF staining in mouse and rat; titrated inhouse to define the optimal dilution (1:50; 1:100; 1:200; 1:500; 1:800)

2. Secondary antibodies for IF staining (host, antigen, fluorophore, source, Cat#) were used in the following dilutions:

All secondary antibodies were tested in different dilutions for optimal staining (1:50; 1:100; 1:200; 1:500; 1:800)

Goat anti-mouse IgG H&L (Alexa Fluor®555); Abcam; Cat#ab150114; 1:200
Alexa Fluor®488 Streptavidin; Biolegend; Cat# 405235; 1:50
Goat anti-Rabbit IgG (H+L) Cross-Adsorbed Secondary Antibody (Alexa Fluor™ 488); Invitrogen; Cat#A-11008; 1:50

3. Flow cytometry antibodies (antigen, fluorophore, clone, source, Cat#) were used in the following dilutions:

All FC antibodies were validated by the manufacturers for IF staining of mouse tissue/cells. FC antibodies were titrated inhouse for optimal performance (1:50; 1:100; 1:200; 1:400; 1:800; 1:1000; 1:2000).

anti-CD45 APC-eFluor™ 780 (clone 30-F11); eBioscience; Cat#47-0451-82; 1:800
anti-CD45 PerCP/ Cyanine 5.5 (clone 30-F11); BioLegend; Cat#103132; 1:400
anti-CD11b FITC (clone M1/70); BD Pharmingen; Cat#557396; 1:400
anti-CD11b PerCP (clone M1/70); BioLegend; Cat#101230; 1:400
anti-CD11b Brilliant Violet 605™ (M1/70); BioLegend; Cat#101257; 1:400
anti-Ly6G PerCP/ Cyanine 5.5 (clone 1A8); BioLegend; Cat#127616; 1:800
anti-Ly6C PE/ Cyanine 7 (clone HK1.4); BioLegend; Cat#128018; 1:1000
anti-CD115 (CSF-1R) Brilliant Violet 421™ (clone AFS98); BioLegend; Cat#135513; 1:800
anti-Siglec-F PE (clone E50-2440); BD Pharmingen; Cat#562068; 1:400
anti-CD117 (c-Kit) Pacific Blue™ (clone 2B8); BioLegend; Cat#105820; 1:400
anti-CD117 (c-Kit) Brilliant Violet 605™ (clone 2B8); BioLegend; Cat#105847; 1:800
anti-CD49b PE/ Cyanine 7 (clone DX5); BioLegend; Cat#108922; 1:400
anti-FcεRIα PE/ Cyanine 7 (clone MAR-1); BioLegend; Cat#134318; 1:100
anti-CD200R3 PE (clone Ba13); BioLegend; Cat#142206; 1:400
anti-CD3ε PE/ Cyanine5 (clone 145-2C11); BioLegend; Cat#100310; 1:400
anti-CD3ε APC (clone 145-2C11); BioLegend; Cat#100312; 1:400
anti-CD19 APC (clone 1D3); BD Pharmingen; Cat#550992; 1:400
anti-CD335 (NKp46) APC (clone 29A1.4); BioLegend; Cat#137608; 1:400
anti-CD31 Alexa Fluor® 647 (clone MEC13.3); BioLegend; Cat#102516; 1:400
anti-CD61 PE (clone 2C9.G2); BioLegend; Cat#104308; 1:400
anti-CD41 Pacific Blue™ (clone MWReg30); BioLegend; Cat#133932; 1:400
anti-Ly-6A/E (Sca-1) PE/ Cyanine 7 (clone E13-161.7); BioLegend; Cat#122514; 1:400
anti-CD16/CD32 APC (clone 93); eBioscience; Cat#17-0161-82; 1:400
anti-CD34 FITC (clone RAM34); BD Pharmingen; Cat#560238; 1:400
anti-CD45R/B220 PerCP (clone RA3-6B2); BD Pharmingen; Cat#553093; 1:400
anti-CD45R/B220 Brilliant Violet 510™ (clone RA3-6B2); BioLegend; Cat#103247; 1:400
anti-TER-119 APC-efluor™ 780 (clone TER-119); eBioscience; Cat#47-5921-82; 1:400
anti-CD71 FITC (clone RI7217); BioLegend; Cat#113806; 1:400
anti-CD5 PerCP(clone 53-7.3); BD Pharmingen; Cat#553025; 1:200

	anti-CD125 (IL-5Rα) APC (clone REA343); Miltenyi Biotec; Cat#130-118-561; 1:100

Animals and other research organisms

Policy information about studies involving animals; ARRIVE guidelines recommended for reporting animal research, and Sex and Gender in Research

Laboratory animals	Wildtype BALB/cJRj were purchased from Janvier Labs. ΔdblGATA, IL-5tg/ 4get, IL-4Rα knockout, and STAT6 knockout mice were on BALB/c background. Cx3cr1creR26-tdTomato mice were on BL/6 background. Steady-state bone phenotype was determined at an age of 10-12 weeks including male and female mice as the BV/TV in the proximal tibia is comparable in male and female BALB/c mice in the young adult phase (2-6 month). All mice were housed in a specific pathogen–free (SPF) facility with a temperature between 22°C and 23°C, humidity of 50%–60%, and a regulated 12-hour light/dark cycle with free access to normal diet food (ssniff, V1534-000) and water.
Wild animals	The study did not involve wild animals.
Reporting on sex	Age-matched female and male mice were used for steady state bone analysis as the BV/TV in the proximal tibia is comparable in male and female BALB/c mice in the young adult phase (2-6 month).<br>Age-matched female and male mice were also used for STA analyses, because female and male K/BxN mice display similar severity of erosive arthritis.<br>For the sham and OVX experiments, female mice were used.
Field-collected samples	Study did not involve samples collected from the field.
Ethics oversight	All experiments were performed according to the rules and regulations of the animal facility FPZ (Franz-Penzoldt-Zentrum, Erlangen). Animal studies were approved by the ethics committees of the government of Unterfranken (Regierung von Unterfranken, Germany).

Note that full information on the approval of the study protocol must also be provided in the manuscript.

Flow Cytometry

Plots

Confirm that:

☒ The axis labels state the marker and fluorochrome used (e.g. CD4-FITC).

☒ The axis scales are clearly visible. Include numbers along axes only for bottom left plot of group (a 'group' is an analysis of identical markers).

☒ All plots are contour plots with outliers or pseudocolor plots.

☒ A numerical value for number of cells or percentage (with statistics) is provided.

Methodology

Sample preparation	For cell isolation from the bone marrow (BM), the left femur was flushed 3x with 1x PBS. Then, the BM cells were lysed with 3 mL RCL buffer and put through 70 µm cell strainers. Blood was lysed twice with 5 mL RCL buffer and put through 70 µm cell strainer.
Instrument	Flow cytometry was performed on the GalliosTM, Cytoflex S flow cytometer or MoFlo Astrios EQ (all from Beckman Counter).
Software	Flow cytometry data were analyzed by Kaluza 2.1 (Beckman Counter) or FlowJo (BD Biosciences).
Cell population abundance	Prior to the sorting process, the eosinophil population within IL-5tg/4get mice constitutes 30% of the total cell count. Subsequent to sorting, the eosinophil purity reaches a level of 90%.<br>Prior to the negative selection by MACS, the neutrophil population within BALB/c mice constitutes 26% of the total cell count. Subsequent to sorting, the neutrophil purity reaches a level of 75%.
Gating strategy	Gating strategy of eosinophils for FACS: Siglec-F+CD125intCD45+ cells<br>Gating strategy of eosinophils for FC: Siglec-F+CD11b+Ly6GintCD45+<br>Gating strategy of osteoclast precursors: CSF-1R+Ly6ChighCD11b+CD45+ cells<br>Gating strategy of neutrophils: CD11b+Ly6GhighCD45+<br>Gating strategy of mast cells: FcεRIα+C-kit+CD11b-CD45+<br>Gating strategy of basophils: CD200R3+CD49b+C-kit-lymphocyte-CD11b+CD45+<br>Gating strategy of LSK: C-kit+Sca-1+lineage-<br>Gating strategy of multipontent progenitors: C-kit+Sca-1-lineage-<br>Gating strategy of common myeloid progenitors: CD16/CD32intCD34+C-kit+Sca-1-lineage-<br>Gating strategy of granulocyte-macrophage progenitors: CD16/CD32highCD34+C-kit+Sca-1-lineage-<br>Gating strategy of megakaryocyte-erythroid progenitors: CD16/CD32-CD34-C-kit+Sca-1-lineage-<br>Gating strategy of pro-erythroblasts: CD71+Ter-119int<br>Gating strategy of EryA erythroblasts: CD71+Ter119+FSChigh<br>Gating strategy of EryB erythroblasts: CD71+Ter119+FSClow<br>Gating strategy of EryC erythroblasts: CD71-Ter119+FSClow<br>Gating strategy of megakaryocytes: CD41+CD61+CD31+

Gating strategy of B-cells: CD19+CD45+
Gating strategy of B1a cells: CD5+CD19+CD45+B220-
Gating strategy of B1b cells: CD5-CD19+CD45+B220-

☒ Tick this box to confirm that a figure exemplifying the gating strategy is provided in the Supplementary Information.
